# Impact of Chronic Inflammation in Psoriasis on Bone Metabolism

**DOI:** 10.3389/fimmu.2022.925503

**Published:** 2022-06-23

**Authors:** Anja Saalbach, Manfred Kunz

**Affiliations:** Department of Dermatology, Venereology and Allergology, University of Leipzig Medical Center, Leipzig, Germany

**Keywords:** chronic inflammation, psoriasis, bone, metabolism, osteoporosis

## Abstract

Psoriasis is a chronic inflammatory disease of the skin and joints associated with several comorbidities such as arthritis, diabetes mellitus and metabolic syndrome, including obesity, hypertension and dyslipidaemia, Crohn’s disease, uveitis and psychiatric and psychological diseases. Psoriasis has been described as an independent risk factor for cardiovascular diseases and thus patients with psoriasis should be monitored for the development of cardiovascular disease or metabolic syndrome. However, there is mounting evidence that psoriasis also affects the development of osteoporosis, an important metabolic disease with enormous clinical and socioeconomic impact. At present, there are still controversial opinions about the role of psoriasis in osteoporosis. A more in depth analysis of this phenomenon is of great importance for affected patients since, until now, bone metabolism is not routinely examined in psoriatic patients, which might have important long-term consequences for patients and the health system. In the present review, we summarize current knowledge on the impact of psoriatic inflammation on bone metabolism and osteoporosis.

## Bone Metabolism and Osteoporosis

Bone undergoes continuous modeling and remodeling, a process involving the coordinated action of osteocytes, the bone resorbing osteoclasts, and bone-forming osteoblasts (1). Osteocytes originate from osteoblasts and are the most abundant bone cells embedded in the bone. Activation of osteocytes by microcracks and microfractures *via* their mechanosensing capability triggers recruitment of osteoclast precursors to the specific bone site and subsequent osteoclast differentiation and bone resorption. Bone remodeling starts with the induction of a resorption cavity by activated osteoclasts (2). Osteoclasts are multinucleated giant cells differentiating from the monocyte/macrophage lineage in the presence of monocyte/macrophage colony–stimulating factor (M-CSF) and the receptor activator of nuclear factor κB (NF-κB) ligand (RANKL) (2). In the course of bone resorption, products of osteoclasts induce recruitment of mesenchymal stem cells (MSCs) and/or osteoprogenitors. After differentiation into osteoblasts, these bone forming cells start to produce bone extracellular matrix including collagen type I, osteocalcin, proteoglycans, bone sialoprotein and osteopontin until the resorbed bone is entirely replaced by a new one. Finally, osteoblasts enclosed in the new bone matrix differentiate into osteocytes. Mineralization of the osteoid completes the bone-remodeling cycle. In healthy bone remodeling, a well-balanced activity of osteoblasts and osteoclasts is controlled by coordinated signaling mechanisms.

The continuous building and degradation of bone can be disturbed by many factors such as hormones and pro-inflammatory factors that interfere with osteoblast function or osteoclast activity. Hyperactivity of osteoclasts or insufficient activity of osteoblasts results in bone loss— known as osteopenia — which finally leads to osteoporosis and enhanced bone fragility (3). Osteoporosis is associated with reduced bone mineral density (BMD), impaired bone microarchitecture, and decreased strength resulting in increased bone fragility. Estrogen deficiency and age-related changes are the cause of primary osteoporosis (3). Estrogen deficiency in post-menopausal women causes osteoporosis in about 50% of women (3). Interestingly, both bone resorption and bone formation are increased in postmenopausal osteoporosis, but the enhanced bone resorption surpasses that of increased bone formation resulting in a net bone loss (4). In contrast, aging is associated with a reduction of both bone formation and resorption. The overall consequences of these age-related changes are cortical thinning, enhanced cortical porosity, thinning of the trabeculae and loss of trabecular connectivity, all of which reduce bone quality and finally the strength of the bone (3, 5). In addition, vitamin D or calcium deficiency, reduction of physical activity, or therapeutic interventions like long-term glucocorticoid treatment are related to secondary osteoporosis (4). Inflammation has been identified as a potential risk factor for osteoporosis. Inflammation is characterized by the activation of cells of the innate and adaptive immune system resulting in the production of inflammatory cytokines perpetuating inflammation but also supports bone degradation and inhibit bone formation. Indeed, a relationship between inflammation and bone disease has been observed in a variety of chronic inflammatory diseases such as rheumatoid arthritis, spondylarthropathies, periodontal diseases, inflammatory bowel disease, coeliac disease, chronic lung inflammation including asthma, chronic obstructive pulmonary disease or alveolitis, and renal diseases (6). Animal studies showing systemic bone loss in experimental models of inflammation supported these data. Bone loss may be mediated by direct effects of inflammation (7, 8). In addition, poor nutrition, catabolic state of inflammatory diseases, reduced body mass, decreased physical activity and long-term glucocorticoid treatment may contribute to inflammation-associated bone loss (6). In a patient population with recent-onset rheumatoid arthritis, increased bone loss was clearly associated with the inflammatory activity of the disease demonstrating the impact of inflammation itself on bone metabolism (9). Epidemiological studies demonstrate that even a small rise in the degree of systemic inflammation can stimulate bone loss and thus, may be an independent risk factor for fractures (9).

Since many cytokines involved in the regulation of bone metabolism also underlie the inflammatory background of psoriasis, we summarize current data on the impact of psoriatic inflammation on bone loss.

## Clinical Features and Pathogenesis of Psoriasis

Psoriasis is a chronic inflammatory skin disease affecting over 60 million adults and children worldwide (10, 11). Skin manifestations are variable, but in classical cases include red, scaly plaques particularly on the extensor surfaces of elbows, knees and on the scalp. But there are also pustular variants that display localized or generalized pustulosis, with a quite different clinical behavior and relative treatment resistance compared to plaque-type variants. Moreover, a significant proportion (20%) of the psoriasis patients suffer from arthritis, which characteristically affects the distal extremities but also big joints (10). In recent years, a substantial number of reports have shown that psoriasis may be regarded as a systemic disease (12–14).

The underlying pathogenesis of psoriasis has many overlaps with other chronic inflammatory diseases such as Rheumatoid arthritis, Crohn’s disease and Lupus erythematosus, and it is currently discussed whether psoriasis may also be regarded as an autoimmune disease. Indeed, it shares many features with other autoimmune diseases such as mechanisms of chronic inflammation, a major role of TNFα in its pathogenesis and an involvement of gene loci similar to those of other autoimmune diseases (15, 16). A candidate autoantigen has recently been identified for psoriasis but its clinical relevance is not completely clear (17).

Psoriasis is a polygenic disease with a strong genetic background, which is in many ways linked to the immunophenotype (15). The evidence for the genetic background came from the observation that siblings of psoriasis patients have a several fold enhanced risk of disease development compared to siblings of non-affected persons (18). In earlier studies, genetic associations were described for the major histocompatibility (MHC) locus on chromosome 6, which harbors the human leukocyte antigen (HLA) genes and a number of other immune genes such as TNFα (19). A strong association was observed for the HLA-C allele Cw6 (HLA-Cw6 is present in almost half of all patients while only in less than 10% of the control population). In more recent genome-wide association studies, a number of additional psoriasis risk variants in common alleles of the general population were identified (20–24). Among significant associations were single nucleotide polymorphisms (SNPs) in the MHC class I region, in genes encoding for IL12B, IL23A and IL23R, IL2/IL21, TNFAIP3 and TNIP1, ZNF313, and epidermal/antimicrobial genes such as SLC12A8, HBD (human β-defensin gene) and the LCE (late cornified envelope) gene cluster. These findings support a role of the TNFα and IL-23/IL-17 pathways for psoriasis pathogenesis. TNFa-induced protein 3 (TNFAIP3) and TNFAIP3-interacting protein 1 (TNIP1) interact with the nuclear factor kappa B (NF-kB) pathway, that plays a central role in mediating TNFα mediated inflammatory signals.

**Figure 1:**
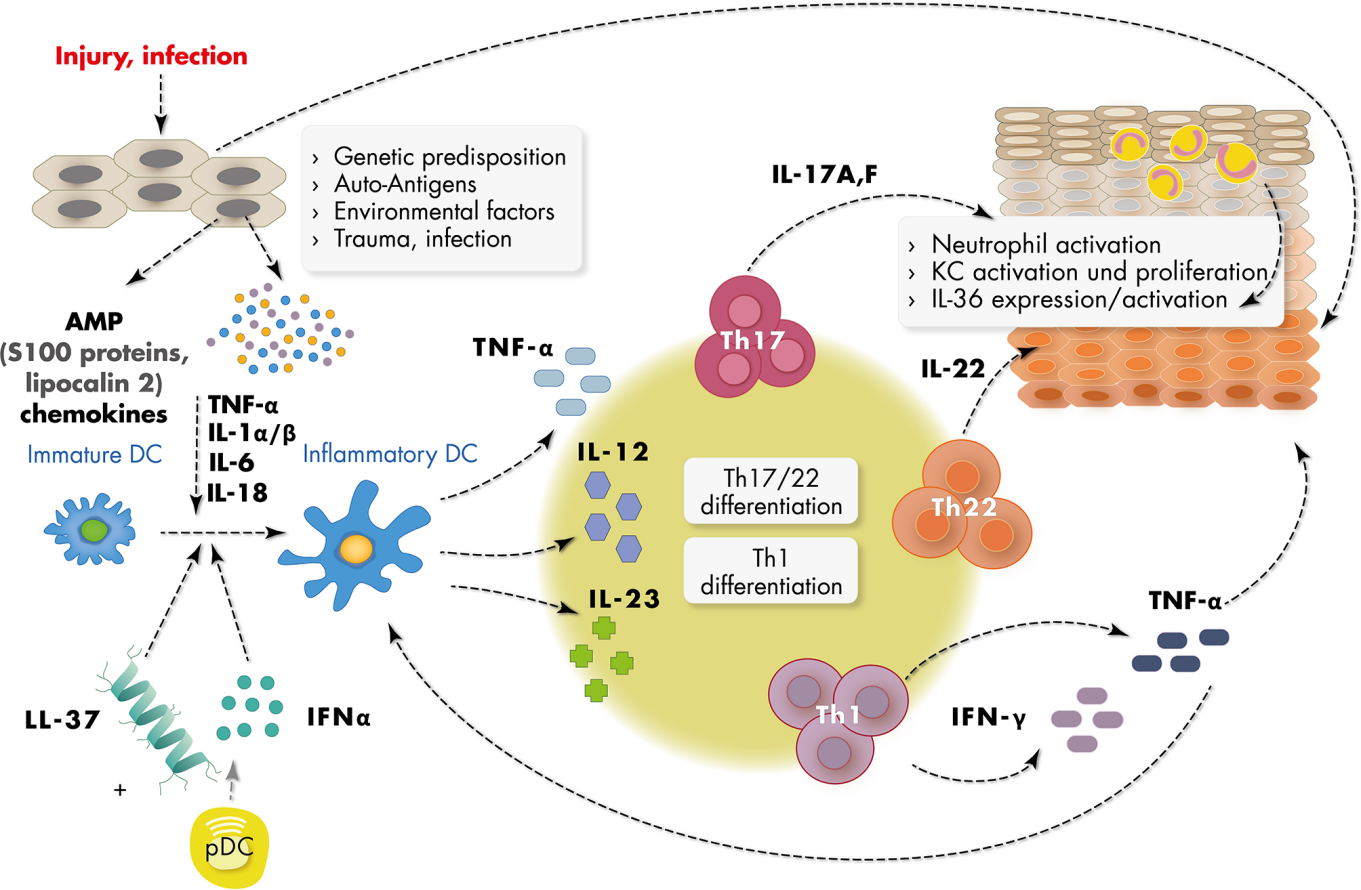
Schematic representation of major inflammatory mediators in psoriasis pathogenesis. Epidermal keratinocytes (KC) in psoriasis are targeted by multiple triggers of the environment such as infection, trauma, traction forces and chemicals and are subsequently activated to produce short nucleotides and antimicrobial peptides (AMPs) such as LL37 and S100 proteins, and chemokines. Activated dermal plasmacytoid dendritic cells (pDC) produce IFN-α in the context of a number of other cytokines such as IFN-γ, TNF-α, and IL-1α/β, which leads to activation of myeloid (inflammatory) dendritic cells. Activated dendritic cells produce cytokines such as IL-23 inducing Th1 and Th17 and Th22 differentiation of T cells. IL-17, IL-22 and IFN-γ produced by these cells further activate keratinocytes. Activated keratinocytes show high proliferation rates and disturbed differentiation leading to epidermal acanthosis and support the inflammatory reaction.

The pathogenesis of psoriasis is based on an interaction between different cell types such as epidermal keratinocytes, antigen-presenting cells and T cells which constitute a pro-inflammatory environment (25, 26). The initiation phase of psoriasis is not completely understood, but a number of reports have emphasized the role of antimicrobiotic peptides (AMP) and dendritic cells in the initial phase of the immune reaction (10, 27, 28). Epidermal keratinocytes in psoriasis are primary targets of multiple triggers of the environment such a traction forces, chemicals and microbes and are activated to produce a number of pro-inflammatory molecules including short nucleotides and antimicrobial peptides. Subsequently plasmacytoid dendritic cells are activated in the dermal compartment to produce IFN-α in the context of a number of other cytokines such as IFN-γ, TNFα, and IL-1β, which leads to activation of myeloid dendritic cells (**Figure 1**). The downstream cascade of cytokines includes IL-23, IL-17, IL-22 and IFNγ, which further activate keratinocytes. Activated keratinocytes show high proliferation rates and further increase the production of a number of pro-inflammatory chemokines such as CXCL1, CXCL8, and CCL2 and AMP such as S100A7/8/9, human β-defenin 2 (hBD2), lipocalin 2 (Lcn2), and the cathelicidin LL37 (29).

The putative pathogenic role of the microbiota (the sum of all microbes that collectively inhabit a given ecosystem) has been described in a number of chronic inflammatory and other diseases (30). Based on current knowledge, the skin and gut microbiota appear to be involved in psoriasis pathogenesis as described by a number of recent reports (31–33). In one report, using 16S rRNA sequencing for the analysis of the skin microbiota, there was an increased abundance of the phylla *Firmicutes* and reduced abundance of *Acinetobacter* in lesional skin compared to control skin (32). The relation changed during treatment. Similar findings were mentioned in a recent review article (34). Moreover, regarding the genus level, the most significant findings were increased amounts of *Prevotella* and *Staphylocoocus* in lesional compared to non-lesional skin (32). Although performed with a limited number of patients, this study provided significant evidence for a role of the microbiota in psoriasis. This might open future perspectives for innovative treatment approaches as skin-targeted bacteriotherapy directed against *Staphylococcus aureus* overrepresentation in skin lesions has successfully been tested in atopic dermatitis (35). In another, more recent study using metagenomic shotgun sequencing, which allows a broader differentiation regarding bacterial diversity compared to classical 16S rRNA sequencing, it was shown that the overall richness of metagenomic bacterial species of the gut microbiota was lower in patients with psoriasis compared to controls (33). Two species of the genus *Blautia* were more prevalent in psoriasis compared to healthy controls. Moreover, evidence was provided for gut metabolic modules active in psoriasis lesions. Taken together, these recent studies suggest that the skin and gut microbiota play a role in psoriasis pathogenesis. However, larger studies and in depth molecular analyses of the direct pathogenic mechanisms are still needed.

Based on current knowledge, the most important and targetable inflammatory mediators include the cytokines TNFα, IL-23 and IL-17, with the knowledge about TNFα being much larger than that of the IL-23/IL-17 axis because of earlier discovery and long-term usage as target for antibody treatment (**Figure 1**). IL-23 itself supports the development of IL-17-secreting CD4^+^ memory T cells (Th17 cells) which then affect other cell types. These findings have led to the recent development of highly effective antibody treatment (biologics) targeting TNFα, IL-23 and IL-17, or downstream pathways, with an ever growing list of new inhibitory antibodies (10). Since a common feature of many of the active inflammatory pathways in psoriasis use the Janus kinase/Signal transducer and activator of transcription (JAK/STAT) system, more recent studies take advantage of these findings to establish small molecule JAK inhibitors for treatment of psoriasis (36). Although data from phase-III clinical trials are promising so far, no JAK inhibitor has been approved from treatment of this disease.

Taken together, pathogenic mechanisms observed in psoriasis involve a number of different cytokines of the T cell immune response and TNFα signaling.

## Pathogenic Relations of Bone Metabolism and Chronic Inflammation in Psoriasis

**Figure 2:**
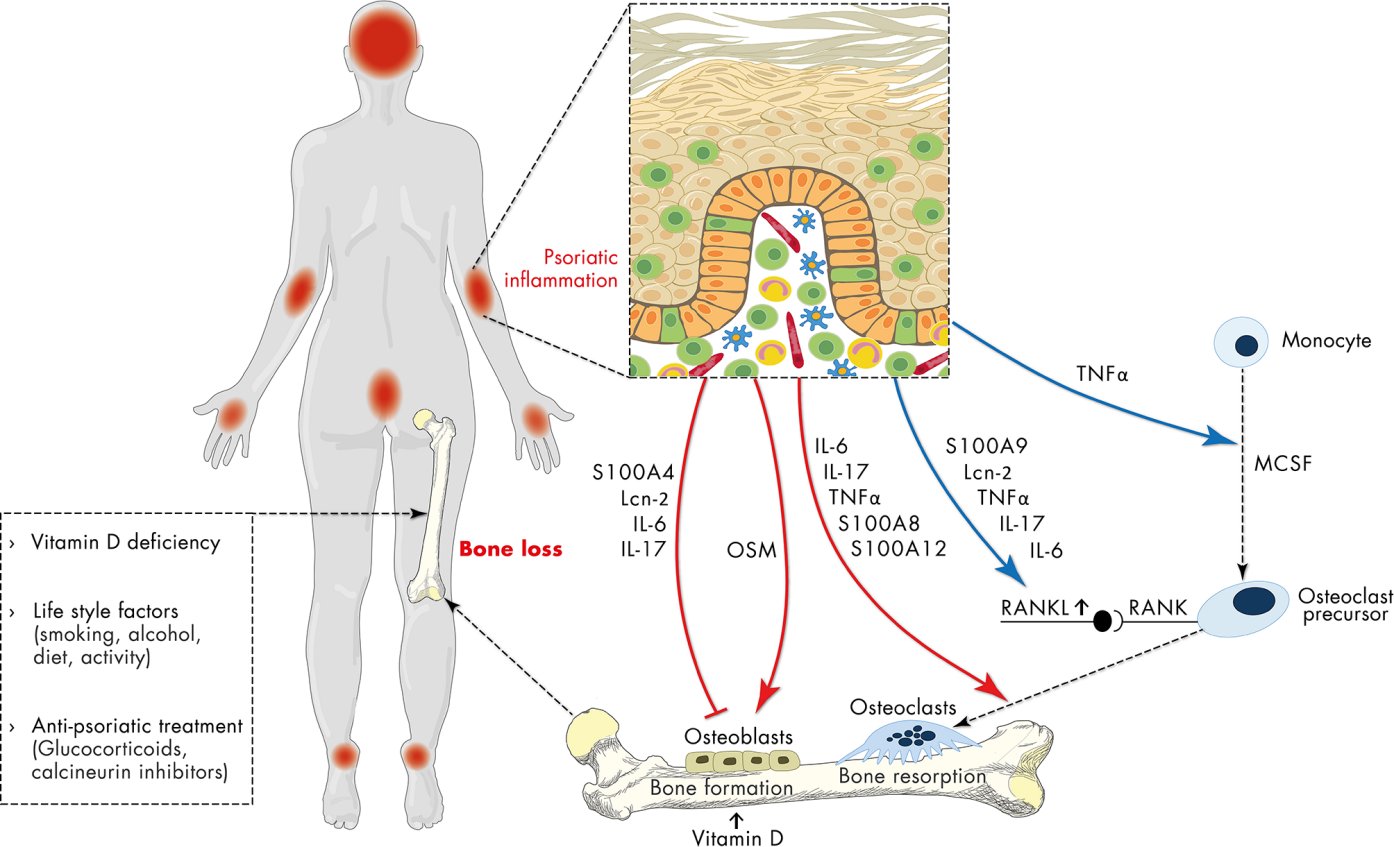
Schematic representation of the relationship of psoriasis and osteoporosis. Apart from vitamin deficiency, life-style factors and anti-psoriatic treatment with e.g., glucocorticoids, the sustained and uncontrolled inflammatory response in psoriasis has a negative impact on bone metabolism contributing to osteoporosis. Psoriasis-associated factors including antimicrobial peptides (S100 proteins, lipocalin 2 (Lcn2)), IL-6, IL-17 directly inhibit osteoblast differentiation, while oncostatin M (OSM) promotes it. On the other hand, psoriasis-associated factors directly support RANKL-induced osteoclast differentiation and indirectly promote osteoclast differentiation by stimulation of RANKL expression. In addition, sustained expression of pro-inflammatory mediators, antimicrobial peptides and chemokines initiates a vicious cycle of inflammation which in turn interferes with both osteoblast and osteoclast differentiation and may thereby connect chronic psoriatic inflammation to impaired bone metabolism.

Dysregulation of the complex interplay between epidermal keratinocytes, antigen-presenting cells and inflammatory T cells promotes the proliferation and attenuates the differentiation of epidermal keratinocytes resulting in the thickened, hyperproliferative epidermis characteristic for psoriatic plaques (10, 28, 37, 38). Importantly, the sustained and uncontrolled inflammatory response in psoriasis is not restricted to the skin. Associations of psoriasis with comorbidities such as cardiometabolic disease, stroke, and metabolic syndrome and the underlying mechanisms have been reviewed in detail in a number of recent reports (11, 39, 40). In psoriasis, a plethora of alarmins (e.g., AMP, DNA fragments), adipokines, pro-inflammatory cytokines and chemokines, as detailed above, conduct the interplay between immune and non-immune cells. A meta-analysis of 63 studies containing 2876 psoriasis patients and 2237 healthy controls showed an increase of a multitude of cytokines in patients with psoriasis (41). Many of these mediators might be able to interfere with both osteoblast and osteoclast differentiation, activation and function and therefore might connect psoriatic inflammation and bone metabolism (**Figure 2**). Indeed, stimulation of skin organ cultures with TNFα, IL-17, osteopontin, or IL-33, cytokines known to be central in the pathogenesis of psoriasis stimulated the expression of pro-osteoclastogenic factors in the skin that in turn promoted the differentiation of monocytes into osteoclast precursors (42).

### Antimicrobial Peptides

As mentioned above, keratinocytes within psoriatic skin lesions produce a wide variety of AMP including cathelicidin, β-defensins, S100 proteins (S100A7, A8, A9 and A12), RNase 7, lysozyme, elafin, and neutrophil gelatinase-associated lipocalin 2 (43). Epidermal expression of S100A8 and S100A9 is a hallmark of psoriasis. Serum levels of S100A8/S100A9 correlate with disease activity in psoriasis patients (44). Moreover, S100A8 and S100A9 are located in the psoriasis susceptibility locus 4 (45). Toll-like receptor 4 (TLR4) has been described as one receptor. Binding of S100A8/A9 to TLR4 induces an inflammatory response in target cells. Consistently, deletion of S100A9 reduces psoriasis-like inflammation in mice (46). In addition to its antimicrobial action, AMP display chemotactic and angiogenic function, and regulate cell proliferation (47). Expression of several S100 proteins such as S100A4, A7, A8, A9 and A12 are highly up-regulated in lesional skin and serum of patients with psoriasis. All of them correlated with disease activity (48). Most of them are able to modify the fine-tuned balance of osteoblast-osteoclast activation and thus might contribute to bone loss during chronic psoriatic skin inflammation (**Figure 2**). S100 proteins might also indirectly interfere with the fine-tuned balance of osteoblast-osteoclast activation by induction of inflammation. S100A8/A9 stimulate the production of pro-inflammatory factors and matrix metalloproteinases (MMPs) in different cell types including endothelial cells and monocytes/macrophages (49). In addition, direct effects of S100 proteins on osteoblasts and osteoclasts have also been described. In detail, extracellular S100A4 inhibits mineralization activity of osteoblasts *via* a receptor for advanced glycation end products (RAGE)-dependent activation of the NF-κB signaling pathway (50). Promotion of osteoclast differentiation from human monocytes by S100A12 and stimulation of osteoclast formation by S100A8 have been described earlier (51, 52). S100A9 stimulates directly the differentiation of monocytes to osteoclasts as well as indirectly by induction of IL-6 and RANKL production *via* engagement with RAGE and TLR4 signaling in osteocytes (53, 54)

Lipocalin 2 (Lcn2) is an antimicrobial protein as well as adipokine associated with obesity and insulin resistance. Serum Lcn2 levels and tissue Lcn2 expression are elevated in psoriatic patients and correlate with the severity of itching (55, 56). Lcn2 is highly expressed in psoriatic skin by neutrophils and keratinocytes driving the infiltration of neutrophils and formation of neutrophil extracellular traps (NET). In an imiquimod-induced mouse model of psoriatic inflammation, application of Lcn2 exacerbated erythema and scaling and increased the gene expression of pro-inflammatory cytokines, chemokines and the cytokines of the IL-23/IL-17 axis (57). In addition to its pro-inflammatory action, Lcn2 has been identified as a mechanical loading sensor. Its expression increases with resting while physical activity reduces its expression. Lcn2 overexpression inhibits osteoblast differentiation and stimulates the production of IL-6 and RANKL, which in turn supports osteoclastogenesis (58). However, Lcn2 levels were unchanged in osteoporotic patients compared to healthy subjects and did not correlate with BMD (59). In contrast, Lim et al. observed that serum Lcn2 levels predict future risk of osteoporotic fractures (60).

### TNFα

TNFα is produced by macrophages, T cells, dendritic cells (DC), neutrophils, as well as by non-immune cells such as fibroblasts. It provides a pro-inflammatory microenvironment allowing an appropriate adaptive immune response. Indeed, the implementation of TNFα blockers into the clinic revolutionized the management of psoriasis and other chronic inflammatory diseases (61). In addition to its pro-inflammatory action, TNFα promotes bone resorption either directly or by stimulating RANKL and macrophage colony-stimulating factor (M-CSF) expression in osteoblasts, stromal cells and osteocytes (8). Blocking TNFα results in reduction of RANKL-positive osteocytes and osteoclast formation in diabetic rats in a model of periodontitis (62, 63). TNFα does not induce osteoclastogenesis alone, but it cooperatively supports osteoclast differentiation from bone marrow-derived macrophages *via* the NF-κB pathway in the presence of RANKL (63).

### IL-6 Cytokine Family

The IL-6 cytokine family includes a variety of cytokines such as IL-6 and oncostatin M (OSM) (64, 65). Classical IL-6 signaling is induced by IL-6 binding to the membrane-bound IL-6 receptor, triggering the association with the signal-transducing gp130 receptor subunit and formation of a heterohexameric complex consisting of IL-6, IL-6R, and gp130. OSM binds with low affinity to gp130 and then recruits either leukemia inhibitory factor (LIF) receptor or OSM receptor. Formation of receptor complexes leads to the activation JAK family of tyrosine kinases and subsequent activation of STAT as well as PI3K-Akt, the p38 and JNK MAPK pathways (66, 67). OSM is mainly produced by haematopoietic cells including T cells, monocytes, macrophages, DC, neutrophils, eosinophils and mast cells, while IL-6 is produced by a broad range of both haematopoietic and nonhaematopoietic cells (66, 67). Skeletal abnormalities including craniosynostosis and progressive scoliosis in patients with missense mutations of gp130 reflect the importance of gp130 signaling during skeleton development (66). Consistently, genetic deletion of gp130 in osteoblasts results in reduced trabecular bone mass, suggesting that cytokines interacting with gp130 may play an important role in bone metabolism (66).

IL-6 is a pleiotropic proinflammatory cytokine and the most abundant cytokine in the circulation. IL-6 is significantly elevated in psoriasis and serum IL-6 correlates with psoriasis area and severity index (PASI) scores in patients with psoriasis (64). It is expressed by keratinocytes, fibroblasts, endothelial cells, DC, macrophages, and Th17 cells and exerts pleiotropic effects including support of keratinocyte growth, stimulation of cytokine and chemokine production by keratinocytes, macrophages and DC, differentiation of Th17 cells, increase of adhesion molecule expression on endothelial cells, and promotion of neutrophil differentiation (68). However, blocking of IL-6 does not improve psoriatic skin inflammation neither in genetic mouse models of psoriatic inflammation nor in patients (69). In contrast, successful therapeutic approaches for various rheumatic diseases by targeting the IL-6 pathway underlines the importance of IL-6 in bone metabolism (70). IL-6 acts on several levels on bone remodeling resulting in a net bone loss. It is able to 1) directly promote osteoclastogenesis, 2) induce secretion of pro-osteoclast mediators like RANKL, IL-1, parathyroid hormone-related protein, and prostaglandin E2 (PGE2) and 3) facilitates osteolysis through inhibition of osteoblast differentiation (71). Consistently, IL-6 knock out mice display a phenotype of increased bone mass (72). In humans, serum IL-6 levels are highly predictive of femoral bone loss in the first decade after the menopause and *IL6* gene polymorphisms are associated with bone mineral density (8, 73).

OSM, mainly secreted by T-cells, monocytes/macrophages, DCs, and neutrophils exerts pro-inflammatory functions by stimulation of AMP and chemokine expression and diminishes keratinocyte differentiation. Overexpression of OSM by intradermal injection of adenovirus vectors induces skin inflammation including leukocyte accumulation, increased expression of pro-inflammatory cytokines, chemokines and AMP, epidermal hyperplasia, keratinocyte proliferation, and inhibits keratinocyte differentiation (74). In humans, OSM is up-regulated in inflammatory skin diseases associated with chronic itch such as psoriasis, atopic dermatitis and cutaneous T cell lymphoma. Consistently, OSM is upregulated in mouse models of atopic dermatitis and psoriasis (65). However, OSM is not required for psoriasis-like imiquimod-induced skin inflammation since genetic deletion of OSM did not affect the psoriatic phenotype in this setting (74).

Several mouse models prove that OSM promotes both bone anabolism and catabolism (75). In detail, mice with global deletion of OSM receptor display higher trabecular bone mass, which was associated with reduced numbers of osteoclasts as well as decreased bone formation suggesting that OSM stimulates both bone resorption and bone formation (76). Overexpression of OSM in knee joints *in vivo* results in increased numbers of osteoclasts and enhanced juxta-articular bone loss caused by increased RANKL expression. On the other hand, application of OSM into the tibia, joint or periosteum stimulates bone formation in mice (76). *In vitro*, OSM supports differentiation of osteoblasts from MSC dependent on OSMR/gp130-activated STAT3 signaling and inhibits sclerostin expression (75). Taken together, OSM is a multifactorial cytokine, highly expressed in psoriatic skin and exerts both anabolic and catabolic effects on bone. The impact of OSM in psoriasis-induced bone loss is a still open question.

## IL-23/IL-17 Axis

One hallmark of psoriasis is the up-regulation of IL-17A in the skin. A significant positive correlation between IL-17 serum levels and disease activity in psoriasis has been shown (77). IL-23 produced upon activation of dendritic cells induces the development of IL-17- and IL-22-secreting CD4+ T cells. Keratin 17, the antimicrobial peptide LL37 and the melanocytic autoantigen ADAMTSL5 are discussed as auto-antigens in psoriasis (17, 78, 79). IL-17 and IL-22 induce epidermal hyperproliferation, neutrophil attraction into the skin, and activation of keratinocytes to produce chemokines and AMP that in turn promote a sustained pathogenic immune cell infiltration (61). IL17- and IL-22-dependent induction of psoriasis-like skin inflammation upon injection of IL-23 into mouse ears highlights the role of IL-23, IL-17 and IL-22 as master regulators in psoriasis (80, 81). The strong reduction of disease activity in psoriatic patients upon blocking IL-17 by neutralizing antibodies highlights the outstanding role of IL-17 in the pathogenesis of psoriasis. In addition, there is substantial evidence that IL-17 is involved in the development of comorbidities in psoriasis (82).

Recently, Uluckan et al. described a pivotal role of IL-17 in psoriatic inflammation induced bone loss (83). They observed lower bone volume and less bony trabeculae in patients with psoriasis without joint involvement compared to healthy controls. Consistently, osteocalcin (OCN) and procollagen type 1 N-terminal propeptide (P1NP), serum markers for bone formation were also shown to be reduced in these patients, while markers for bone resorption such as C-terminal telopeptide of type 1 collagen (CTX) or the RANKL/osteoprotegerin (OPG) ratio were not affected. These data were confirmed by an own study showing that patients with psoriasis have decreased serum P1NP level independent of age, sex and weight while CTX-I was unaffected (84). Importantly, serum IL-17A levels were inversely correlated to bone volume and serum OCN and P1NP levels (85). The relevance of IL-17A in control of bone metabolism has also been emphasized in post-menopausal women suffering from osteoporosis. These patients showed elevated serum concentrations of IL-17A, which correlated negatively with bone mineral density (86). These data were confirmed in different genetic mouse models of psoriatic inflammation as well as in the imiquimod-induced psoriatic inflammation model (84, 85). Cell differentiation and mineralization assays on calvarial and bone marrow stromal cells in the presence of recombinant IL-17A showed that IL-17A reduces the mineralization capacity and expression of osteoblast differentiation marker by inhibiting the Wnt signaling pathway in osteoblasts and osteocytes. Rescue of bone loss and Wnt target gene expression upon blocking of IL-17A in a genetic mouse model of psoriatic inflammation, supports the pathogenic role of IL-17 in inflammation-induced bone loss (85). Consistently, ovariectomy-induced bone loss is prevented in IL-17 receptor deficient mice or upon blocking IL-17 (85, 87).

Regarding IL-17 effects on osteoclasts data are controversially discussed (88). *In vivo* gene transfer of IL-17A *via* tail vein injection resulted in systemic IL-17 expression. In this setting, IL-17 increased response of precursors cells to M-CSF and RANKL by stimulation M-CSF-R and RANK expression and enhanced serum tartrate-resistant acid phosphatase (TRAP), resulting in bone destruction as detected by micro-computed tomography (CT) (89). Yago et al. describe a stimulatory effect of IL-17 on osteoclastogenesis (90). In contrast to the data described above (85), other studies did not show any effect of IL17 on osteoclast development (91). At least the discrepancy in the *in vitro* studies might be explained by the different source of precursor cells used in these studies. Sprangers et al. demonstrated that the different monocyte subtypes used as a source for osteoclast precursors in these studies are able to differentiate into osteoclasts *in vitro* but osteoclastogenesis and subsequent bone resorption was distinctly affected by IL-17A (92).

Taken together, psoriasis is accompanied by attenuation of bone metabolism *via* IL-17. These data also suggest that blocking IL-17 might reverse inflammation-induced bone loss in addition to the improvement of the skin manifestations in patients with psoriasis. However, until now, bone quality is not routinely assessed in psoriatic patients and data about bone loss in patients with psoriasis before and after treatment with IL-17 blocking antibodies are still lacking.

## Vitamin D Deficiency in Psoriasis

Vitamin D deficiency is a main risk factor of both psoriasis and osteoporosis. Vitamin D3 is naturally synthesized in the epidermis from its precursor 7-dehydrocholesterol upon the action of UVB after sun exposure. Keratinocytes express CYP27A1 and CYP27B1 enzymes that convert the pre-vitamin D3 into its active form, 1,25(OH)_2_ vitamin D_3_ that is also released into the circulation (93, 94)

Vitamin D regulates the metabolism of calcium and phosphate as well as parathyroid hormone (PTH) secretion and is thus indispensable for the maintenance of bone integrity. Vitamin D deficiency results in an imbalance in the calcium/phosphate ratio and finally leads to bone mineralization disorders such as osteoporosis. The central role of vitamin D in bone turnover is reflected by the support of anti-osteoporotic therapies *via* supplementation of vitamin D (94). Vitamin D deficiency in psoriatic patients and an inverse correlation between serum 25(OH) vitamin D and disease severity has been reported in several studies (57, 95). In line with this, keratinocytes within psoriasis lesional skin express lower levels of CYP27A1 and CYP27B1 (96). In addition, patient’s behavior such as covering psoriatic skin for cosmetic reasons might cause lower sun exposure and vitamin D production. Thus, vitamin D deficiency in psoriasis might contribute to altered bone metabolism in psoriasis (**Figure 2**).

## Anti-psoriatic Therapies

Anti-psoriatic therapies include topical treatment with glucocorticoids or retinoids and systemic treatment such as methotrexate, cyclosporine, corticosteroids, fumarate and several biologics targeting the key players in psoriasis (TNFα, IL-17 and IL-23) or their signaling pathways.

Among the anti-psoriatic therapies corticosteroids display the strongest impact on bone. The negative effect of systemic glucocorticoid treatment on bone is well known and termed glucocorticoid-induced osteoporosis, the most common secondary cause of osteoporosis. About 30% of all patients treated with systemic glucocorticoids for more than 6 months develop glucocorticoid-induced osteoporosis (97). However, topical treatment with glucocorticoid is widely used in psoriasis (**Figure 2**). A nationwide retrospective cohort study showed that use of high cumulative amounts of topical corticosteroids was associated with an increased risk of osteoporosis and osteoporotic fractures (98).

The effect of methotrexate on bone has not been extensively studied. Recent studies did not find an increased fracture risk in psoriasis patients treated with methotrexate, compared to those who did not receive methotrexate (99). An *in vitro* study demonstrated that methotrexate attenuates the differentiation of osteoblast precursors and thus bone formation (100). However, the anti-inflammatory effect might overwhelm these potential deleterious effects of methotrexate on bone (101).

Like glucocorticoids, calcineurin inhibitors such as ciclosporin A cause severe and rapid trabecular bone loss. It is able to inhibit the osteoblast differentiation *via* calcineurin-nuclear factor of activated T cells (NFAT) signaling. However, the effect of ciclosporin A on bone metabolism has been mostly studied in patients with solid organ transplants (101).

## Life Style Factors

Life style factors such as smoking, physical activity, alcohol consume can affect bone metabolism. Importantly, most of these life style factors are affected in psoriatic patients (**Figure 2**).

*Cigarette smoking* is an important risk factor for osteoporosis. There is a direct relationship between tobacco use and decreased bone density (102). Tobacco affects the calciotropic hormone metabolism, intestinal calcium absorption, sex hormone production, adrenal cortical hormone metabolism, RANK-RANKL-OPG system and displays direct effects on bone cells (102). In addition, smoking indirectly affects bone metabolism since smokers often have less weight, consume more alcohol, are less physically active or have a poor nutrition status. Interestingly, psoriatic patients have a higher incidence of smoking habit as well as an increased consume of cigarettes per day compared with patients without psoriasis (103, 104).

Studies in humans and animals indicate that chronic heavy *alcohol consume* strongly impairs bone quality and may support the development of osteoporosis (105). Alcohol has toxic effects, suppresses osteoblastic differentiation of bone marrow cells and promotes adipogenesis (106). A systematic literature search provided evidence that alcohol consumption is greater in psoriasis patients than in the general population (107).

*Physical activity* is a powerful stimulus for bone generation and metabolism. Mechanical stress mediated by muscle contraction and exercise increases bone density. Inactive bone such as during gravidity loss during space flight, after spinal cord injury, or prolonged bed rest, leads to loss of bone mass (105). Patients with psoriasis exhibit decreased total physical activity. A significant negative correlation between physical activity and disease severity as well as life quality index has been observed in psoriasis patients (108). A survey of hospitalized patients with psoriasis revealed that patients with psoriasis have a decreased physical exercise and lower-intensity activity compared to healthy controls. Psoriasis itself may lead to either physical impairment and/or a decrease in life quality (109). Lower physical activity might contribute to the development of obesity, cardiovascular disease or decreased bone formation in psoriasis.

## Epidemiological Association of Bone Metabolism and Chronic Inflammation in Psoriasis

The high expression of pro-inflammatory mediators in psoriasis and their crucial role in bone remodeling suggests an involvement of these mediators in inflammation-related bone loss. Although there are controversial data regarding the osteoporosis risk in psoriatic patients the majority of studies describe a decreased BMD in patients with psoriasis. In the HUNT3 study including 48,194 participants and few smaller studies, no association between psoriasis and bone fracture risk, BMD or higher prevalence of osteoporosis has been observed in patients with psoriasis (110–115). However, in the last years several large-scale population studies and smaller studies reported a positive correlation (116–122). An association of osteoporosis with a previous diagnosis of psoriasis was observed in a longitudinal study using data of the health insurance database in Taiwan with 17,507 osteoporosis patients and 52,521 controls (116). Consistently, in a large cross-sectional study in the U.S., an association of psoriasis and psoriatic arthritis with osteopenia, osteoporosis, and ankylosing spondylitis has been observed (117). The risk of osteopenia/osteoporosis was dependent on the average duration of psoriatic disease (123). Recently, Lee et al. used two different protocols to evaluate the association of psoriasis and osteoporosis. The group showed in a large scale study using the Korean National Health Insurance that the risk of osteoporosis was higher in psoriasis patients than in control participants. In a second approach they found that the prevalence of psoriasis was significantly higher in osteoporosis patients than in controls (122). A meta analysis showed an increased risk of fractures in patients with psoriasis/psoriatic arthritis (124, 125). Raimondo et al. showed increased serum RANKL levels and osteoclast differentiation as well as activity in monocytes from psoriatic patients with severe skin disease in comparison to those with mild skin disease (42).

## Concluding Remarks

Taken together, emerging evidence suggest that patients with psoriasis, with and without psoriatic arthritis, are at greater risk for osteopenia/osteoporosis. The mechanism may be related to chronic inflammation in psoriasis. However, drugs used for treatment such as corticosteroids, reduced physical activity, and life style can also promote development of osteoporosis in these patients. Epidemiological studies as well as *in vitro* and vivo studies suggest that it might be advisable to monitor BMD in patients with psoriasis.

Evaluation of bone quality in patients with psoriasis before and after treatment with neutralizing antibodies would answer the question of the contribution of specific cytokines to the development of inflammation-induced bone loss in psoriasis. There is an ongoing clinical trial, (METABOLyx trial, EudraCT no. 2016-001671-79, NCT03440736), addressing this question. An exploratory biomarker sub-study determines bone metabolism serum markers such as P1NP, CTX, RANKL, OPG, sclerostin, sThy-1 at baseline, week 16 and week 28 during treatment of 50 patients with psoriasis with secukinumab (126). This study will give first hints whether targeting IL-17 alters bone metabolism in addition to its great impact on skin inflammation. Since only overweight patients are included, the data might be difficult to interpret.

Regarding the great impact of osteoporosis on morbidity, mortality and life quality of patients as well as on socioeconomic factors, further studies are needed to define this issue to find the best treatment for skin and bone disease.

## Author Contributions

All authors listed have made a substantial, direct, and intellectual contribution to the work and approved it for publication.

## Funding

This work was supported by the PsoNet Leipzig/Westsachsen and the Hautnetz. The authors acknowledge support from the University of Leipzig within the program of Open Access Publishing.

## Conflict of Interest

MK received travel grants from UCB Pharma, and is member of advisory boards of Novartis Pharma and LEO Pharma.

The remaining author declares that the research was conducted in the absence of any commercial or financial relationships that could be construed as a potential conflict of interest.

## Publisher’s Note

All claims expressed in this article are solely those of the authors and do not necessarily represent those of their affiliated organizations, or those of the publisher, the editors and the reviewers. Any product that may be evaluated in this article, or claim that may be made by its manufacturer, is not guaranteed or endorsed by the publisher.

## References

[1] Shahi M Peymani A Sahmani M. Regulation of Bone Metabolism. Rep Biochem Mol Biol (2017) 5(2):73–82. doi: 10.1201/9781420028836.ch14 28367467 PMC5346273

[2] Greenhill C. Function of Soluble RANKL Deciphered. Nat Rev Endocrinol (2019) 15:628. doi: 10.1038/s41574-019-0264-9 31519976

[3] Reid IR. Revisiting Osteoporosis Guidelines. Lancet Diabetes Endocrinol (2021) 9:805–6. doi: 10.1016/S2213-8587(21)00283-7 34688355

[4] Feng X McDonald JM. Disorders of Bone Remodeling. AnnuRevPathol (2011) 6:121–45. doi: 10.1146/annurev-pathol-011110-130203 PMC3571087 20936937

[5] Demontiero O Vidal C Duque G. Aging and Bone Loss: New Insights for the Clinician. TherAdvMusculoskeletDis (2012) 4:61–76. doi: 10.1177/1759720X11430858 PMC3383520 22870496

[6] Hardy R Cooper MS. Bone Loss in Inflammatory Disorders. J Endocrinol (2009) 201:309–20. doi: 10.1677/JOE-08-0568 19443863

[7] Polzer K Joosten L Gasser J Distler JH Ruiz G Baum W. Interleukin-1 is Essential for Systemic Inflammatory Bone Loss. AnnRheumDis (2010) 69:284–90. doi: 10.1136/ard.2008.104786 19196726

[8] Redlich K Smolen JS. Inflammatory Bone Loss: Pathogenesis and Therapeutic Intervention. Nat Rev Drug Discovery (2012) 11:234–50. doi: 10.1038/nrd3669 22378270

[9] Guler-Yuksel M Hoes JN Bultink IEM Lems WF. Glucocorticoids, Inflammation and Bone. Calcif Tissue Int (2018) 102:592–606. doi: 10.1007/s00223-017-0335-7 29313071

[10] Griffiths CEM Armstrong AW Gudjonsson JE Barker JNWN. Psoriasis. Lancet (2021) 397:1301–15. doi: 10.1016/S0140-6736(20)32549-6 33812489

[11] Boehncke W-H Schön MP. Psoriasis. Lancet (2015) 386:983–94. doi: 10.1016/S0140-6736(14)61909-7 26025581

[12] Boehncke WH Gladman DD Chandran V. Cardiovascular Comorbidities in Psoriasis and Psoriatic Arthritis: Pathogenesis, Consequences for Patient Management, and Future Research Agenda: A Report From the GRAPPA 2009 Annual Meeting. J Rheumatol (2011) 38:567–71. doi: 10.3899/jrheum.101124 21362790

[13] Husni ME Wilson Tang WH Lucke M Chandrasekharan UM Brennan DM Hazen SL. Correlation of High-Density Lipoprotein-Associated Paraoxonase 1 Activity With Systemic Inflammation, Disease Activity, and Cardiovascular Risk Factors in Psoriatic Disease. Arthritis Rheumatol (2018) 70:1240–50. doi: 10.1002/art.40499 29569857

[14] Ryan C Korman NJ Gelfand JM Lim HW Elmets CA Feldman SR. Research Gaps in Psoriasis: Opportunities for Future Studies. JAmAcadDermatol (2014) 70:146–67. doi: 10.1016/j.jaad.2013.08.042 24126079

[15] Harden JL Krueger JG Bowcock AM. The Immunogenetics of Psoriasis: A Comprehensive Review. J Autoimmun (2015) 64:66–73. doi: 10.1016/j.jaut.2015.07.008 26215033 PMC4628849

[16] Bowcock AM Cookson WO. The Genetics of Psoriasis, Psoriatic Arthritis and Atopic Dermatitis. HumMolGenet (2004) 13 Spec No:R43–55. doi: 10.1093/hmg/ddh094 14996755

[17] Arakawa A Siewert K Stohr J Besgen P Kim SM Ruhl G. Melanocyte Antigen Triggers Autoimmunity in Human Psoriasis. JExpMed (2015) 212:2203–12. doi: 10.1084/jem.20151093 PMC4689169 26621454

[18] Bhalerao J Bowcock AM. The Genetics of Psoriasis: A Complex Disorder of the Skin and Immune System. HumMolGenet (1998) 7:1537–45. doi: 10.1093/hmg/7.10.1537 9735374

[19] Roberson ED Bowcock AM. Psoriasis Genetics: Breaking the Barrier. Trends Genet (2010) 26:415–23. doi: 10.1016/j.tig.2010.06.006 PMC2957827 20692714

[20] Capon F Bijlmakers MJ Wolf N Quaranta M Huffmeier U Allen M. Identification of ZNF313/RNF114 as a Novel Psoriasis Susceptibility Gene. HumMolGenet (2008) 17:1938–45. doi: 10.1093/hmg/ddn091 PMC2900900 18364390

[21] Ellinghaus D Jostins L Spain SL Cortes A Bethune J Han B. Analysis of Five Chronic Inflammatory Diseases Identifies 27 New Associations and Highlights Disease-Specific Patterns at Shared Loci. NatGenet (2016) 48:510–8.. doi: 10.1038/ng.3528 PMC4848113 26974007

[22] Nair RP Duffin KC Helms C Ding J Stuart PE Goldgar D. Genome-Wide Scan Reveals Association of Psoriasis With IL-23 and NF-kappaB Pathways. NatGenet (2009) 41:199–204. doi: 10.1038/ng.311 PMC2745122 19169254

[23] Stuart PE Nair RP Tsoi LC Tejasvi T Das S Kang HM. Genome-Wide Association Analysis of Psoriatic Arthritis and Cutaneous Psoriasis Reveals Differences in Their Genetic Architecture. AmJHumGenet (2015) 97:816–36. doi: 10.1016/j.ajhg.2015.10.019 PMC4678416 26626624

[24] Zhang XJ Huang W Yang S Sun LD Zhang FY Zhu QX. Psoriasis Genome-Wide Association Study Identifies Susceptibility Variants Within LCE Gene Cluster at 1q21. NatGenet (2009) 41:205–10. doi: 10.1038/ng.310 19169255

[25] Brembilla NC Senra L Boehncke WH. The IL-17 Family of Cytokines in Psoriasis: IL-17A and Beyond. Front Immunol (2018) 9:1682. doi: 10.3389/fimmu.2018.01682 30127781 PMC6088173

[26] Hawkes JE Yan BY Chan TC Krueger JG. Discovery of the IL-23/IL-17 Signaling Pathway and the Treatment of Psoriasis. J Immunol (2018) 201:1605–13. doi: 10.4049/jimmunol.1800013 PMC6129988 30181299

[27] Guttman-Yassky E Krueger JG. Psoriasis: Evolution of Pathogenic Concepts and New Therapies Through Phases of Translational Research. Br J Dermatol (2007) 157:1103–15. doi: 10.1111/j.1365-2133.2007.08135.x 17714560

[28] Nestle FO Kaplan DH Barker J. Psoriasis. N Engl J Med (2009) 361:496–509. doi: 10.1056/NEJMra0804595 19641206

[29] Zhou X Chen Y Cui L Shi Y Guo C. Advances in the Pathogenesis of Psoriasis: From Keratinocyte Perspective. Cell Death Dis (2022) 13:81. doi: 10.1038/s41419-022-04523-3 35075118 PMC8786887

[30] Lynch SV Pedersen O. The Human Intestinal Microbiome in Health and Disease. N Engl J Med (2016) 375:2369–79. doi: 10.1056/NEJMra1600266 27974040

[31] Liang X Ou C Zhuang J Li J Zhang F Zhong Y. Interplay Between Skin Microbiota Dysbiosis and the Host Immune System in Psoriasis: Potential Pathogenesis. Front Immunol (2021) 12:764384. doi: 10.3389/fimmu.2021.764384 34733291 PMC8558530

[32] Langan EA Künstner A Miodovnik M Zillikens D Thaçi D Baines JF. Combined Culture and Metagenomic Analyses Reveal Significant Shifts in the Composition of the Cutaneous Microbiome in Psoriasis. Br J Dermatol (2019) 181(6):1254–64. doi: 10.1111/bjd.17989 30985920

[33] Todberg T Egeberg A Zachariae C Sørensen N Pedersen O Skov L. Patients With Psoriasis Have a Dysbiotic Taxonomic and Functional Gut Microbiota. Br J Dermatol (2022). doi: 10.1111/bjd.21245 35289939

[34] Yerushalmi M Elalouf O Anderson M Chandran V. The Skin Microbiome in Psoriatic Disease: A Systematic Review and Critical Appraisal. J Transl Autoimmun (2019) 2:100009. doi: 10.1016/j.jtauto.2019.100009 32743498 PMC7388378

[35] Nakatsuji T Hata TR Tong Y Cheng JY Shafiq F Butcher AM. Development of a Human Skin Commensal Microbe for Bacteriotherapy of Atopic Dermatitis and Use in a Phase 1 Randomized Clinical Trial. Nat Med (2021) 27:700–9. doi: 10.1038/s41591-021-01256-2 PMC8052297 33619370

[36] Funk PJ Perche PO Singh R Kelly KA FS. Comparing Available JAK Inhibitors for Treating Patients With Psoriasis. Expert Rev Clin Immunol (2022) 10:1–14. doi: 10.1080/1744666X.2022.2039121 35129030

[37] Kim J Krueger JG. The Immunopathogenesis of Psoriasis. Dermatol Clin (2015) 33:13–23. doi: 10.1016/j.det.2014.09.002 25412780

[38] Nickoloff BJ Qin JZ Nestle FO. Immunopathogenesis of Psoriasis. Clin Rev Allergy Immunol (2007) 33:45–56. doi: 10.1007/s12016-007-0039-2 18094946

[39] Fernandez-Armenteros JM Gomez-Arbones X Buti-Soler M Betriu-Bars A Sanmartin-Novell V Ortega-Bravo M. Psoriasis, Metabolic Syndrome and Cardiovascular Risk Factors. A Population-based Study J Eur Acad Dermatol Venereol (2019) 33:128–35. doi: 10.1111/jdv.15159 29953676

[40] Gerdes S Mrowietz U Boehncke WH. [Comorbidity in Psoriasis]. Hautarzt (2016) 67:438–44. doi: 10.1007/s00105-016-3805-3 27221798

[41] Bai F Zheng W Dong Y Wang J Garstka MA Li R. Serum Levels of Adipokines and Cytokines in Psoriasis Patients: A Systematic Review and Meta-Analysis. Oncotarget (2018) 9:1266–78. doi: 10.18632/oncotarget.22260 PMC5787437 29416693

[42] Raimondo A Lembo S Di CR Donnarumma G Monfrecola G Balato N. Psoriatic Cutaneous Inflammation Promotes Human Monocyte Differentiation Into Active Osteoclasts, Facilitating Bone Damage. EurJImmunol (2017) 47:1062–74. doi: 10.1002/eji.201646774 28386999

[43] Christmann C Zenker S Martens L Hübner J Loser K Vogl T. Interleukin 17 Promotes Expression of Alarmins S100A8 and S100A9 During the Inflammatory Response of Keratinocytes. Front Immunol (2021) 11:599947. doi: 10.3389/fimmu.2020.599947 33643287 PMC7906991

[44] Benoit S Toksoy A Ahlmann M Schmidt M Sunderkötter C Foell D. Elevated Serum Levels of Calcium-Binding S100 Proteins A8 and A9 Reflect Disease Activity and Abnormal Differentiation of Keratinocytes in Psoriasis. Br J Dermatol (2006) 155:62–6. doi: 10.1111/j.1365-2133.2006.07198.x 16792753

[45] Semprini S Capon F Tacconelli A Giardina E Orecchia A Mingarelli R. Evidence for Differential S100 Gene Over-Expression in Psoriatic Patients From Genetically Heterogeneous Pedigrees. Hum Genet (2002) 111:310–3. doi: 10.1007/s00439-002-0812-5 12384771

[46] Schonthaler HB Guinea-Viniegra J Wculek SK Ruppen I Ximenez-Embun P Guio-Carrion A. S100A8-S100A9 Protein Complex Mediates Psoriasis by Regulating the Expression of Complement Factor C3. Immunity (2013) 39:1171–81. doi: 10.1016/j.immuni.2013.11.011 24332034

[47] Takahashi T Yamasaki K. Psoriasis and Antimicrobial Peptides. Int J Mol Sci (2020) 21:6791. doi: 10.3390/ijms21186791 PMC7555190 32947991

[48] Wilsmann-Theis D Wagenpfeil J Holzinger D Roth J Koch S Schnautz S. Among the S100 Proteins, S100A12 Is the Most Significant Marker for Psoriasis Disease Activity. J Eur Acad Dermatol Venereol (2016) 30:1165–70. doi: 10.1111/jdv.13269 26333514

[49] Di Ceglie I Kruisbergen NNL van den Bosch MHJ van Lent PLEM. Fc-Gamma Receptors and S100A8/A9 Cause Bone Erosion During Rheumatoid Arthritis. Do They Act as Partners in Crime? Rheumatology (2019) 58:1331–43. doi: 10.1093/rheumatology/kez218 31180451

[50] Kim H Lee YD Kim MK Kwon J-O Song M-K Lee ZH. Extracellular S100A4 Negatively Regulates Osteoblast Function by Activating the NF-κb Pathway. BMB Rep (2017) 50:97–102. doi: 10.5483/BMBRep.2017.50.2.170 27998393 PMC5342873

[51] Grevers LC de Vries TJ Vogl T Abdollahi-Roodsaz S Sloetjes AW Leenen PJM. S100A8 Enhances Osteoclastic Bone Resorption *In Vitro* Through Activation of Toll-Like Receptor 4: Implications for Bone Destruction in Murine Antigen-Induced Arthritis. Arthritis Rheum (2011) 63:1365–75. doi: 10.1002/art.30290 21337316

[52] Nishida M Saegusa J Tanaka S Morinobu A. S100A12 Facilitates Osteoclast Differentiation From Human Monocytes. PloS One (2018) 13(9):e0204140. doi: 10.1371/journal.pone.0204140 30235276 PMC6147475

[53] Takagi R Sakamoto E Kido J Inagaki Y Hiroshima Y Naruishi K. S100A9 Increases IL-6 and RANKL Expressions Through MAPKs and STAT3 Signaling Pathways in Osteocyte-Like Cells. BioMed Res Int (2020) 2020:1–12. doi: 10.1155/2020/7149408 PMC7053464 32149126

[54] Dapunt U Giese T Maurer S Stegmaier S Prior B Hansch GM. Neutrophil-Derived MRP-14 is Up-Regulated in Infectious Osteomyelitis and Stimulates Osteoclast Generation. J Leukoc Biol (2015) 98:575–82. doi: 10.1189/jlb.3VMA1014-482R 25765681

[55] Hassab-El-Naby HM Helal EA Abdou AM Abu Elkheir OA Elsaie ML Ibrahim SM. Evaluation of Tissue and Serum Lipocalin 2 in Psoriasis Vulgaris and its Implications on Subclinical Atherosclerosis. Dermatol Rev (2021) 2:245–50. doi: 10.1002/der2.60

[56] Aizawa N Ishiuji Y Tominaga M Sakata S Takahashi N Yanaba K. Relationship Between the Degrees of Itch and Serum Lipocalin-2 Levels in Patients With Psoriasis. J Immunol Res (2019) 2019:1–8. doi: 10.1155/2019/8171373 PMC6360588 30805373

[57] Hau CS Kanda N Tada Y Shibata S Uozaki H Fukusato T. Lipocalin-2 Exacerbates Psoriasiform Skin Inflammation by Augmenting T-Helper 17 Response. J Dermatol (2016) 43:785–94. doi: 10.1111/1346-8138.13227 26702876

[58] Rucci N Capulli M Piperni SG Cappariello A Lau P Frings-Meuthen P. Lipocalin 2: A New Mechanoresponding Gene Regulating Bone Homeostasis. J Bone Miner Res (2015) 30:357–68. doi: 10.1002/jbmr.2341 25112732

[59] Maurizi A Ponzetti M Gautvik KM Reppe S Teti A Rucci N. Lipocalin 2 Serum Levels Correlate With Age and Bone Turnover Biomarkers in Healthy Subjects But Not in Postmenopausal Osteoporotic Women. Bone Rep (2021) 14:101059. doi: 10.1016/j.bonr.2021.101059 34026950 PMC8121999

[60] Lim WH Wong G Lim EM Byrnes E Zhu K Devine A. Circulating Lipocalin 2 Levels Predict Fracture-Related Hospitalizations in Elderly Women: A Prospective Cohort Study. J Bone Miner Res (2015) 30:2078–85. doi: 10.1002/jbmr.2546 25939604

[61] Mylonas A Conrad C. Psoriasis: Classical vs Paradoxical Yin-Yang TNF Type I Interferon. Front Immunol (2018) 9:2746. doi: 10.3389/fimmu.2018.02746 30555460 PMC6283263

[62] Marahleh A Kitaura H Ohori F Kishikawa A Ogawa S Shen W-R. TNF-α Directly Enhances Osteocyte RANKL Expression and Promotes Osteoclast Formation. Front Immunol (2019) 10:2925. doi: 10.3389/fimmu.2019.02925 31921183 PMC6923682

[63] Luo G Li F Li X Wang Z Zhang B. TNF−α and RANKL Promote Osteoclastogenesis by Upregulating RANK *via* the NF−κb Pathway. Mol Med Rep (2018) 17(5):6605–11. doi: 10.3892/mmr.2018.8698 PMC5928634 29512766

[64] Muramatsu S Kubo R Nishida E Morita A. Serum Interleukin-6 Levels in Response to Biologic Treatment in Patients With Psoriasis. Mod Rheumatol (2017) 27:137–41. doi: 10.3109/14397595.2016.1174328 27194220

[65] Tseng P-Y Hoon MA. Oncostatin M can Sensitize Sensory Neurons in Inflammatory Pruritus. Sci Transl Med (2021) 13:eabe3037. doi: 10.1126/scitranslmed.abe3037 34757808 PMC9595590

[66] West NR Owens BMJ Hegazy AN. The Oncostatin M-Stromal Cell Axis in Health and Disease. Scand J Immunol (2018) 88:e12694. doi: 10.1111/sji.12694 29926972

[67] Hirano T. IL-6 in Inflammation, Autoimmunity and Cancer. Int Immunol (2021) 33:127–48. doi: 10.1093/intimm/dxaa078 PMC7799025 33337480

[68] Blauvelt A. IL-6 Differs From TNF-α: Unpredicted Clinical Effects Caused by IL-6 Blockade in Psoriasis. J Invest Dermatol (2017) 137(3):541–2. doi: 10.1016/j.jid.2016.11.022 28235443

[69] Wang Y Golden JB Fritz Y Zhang X Diaconu D Camhi MI. Interleukin 6 Regulates Psoriasiform Inflammation–Associated Thrombosis. JCI Insight (2016) 1:e89384. doi: 10.1172/jci.insight.89384 27942589 PMC5135273

[70] Choy EH De Benedetti F Takeuchi T Hashizume M John MR Kishimoto T. Translating IL-6 Biology Into Effective Treatments. Nat Rev Rheumatol (2020) 16:335–45. doi: 10.1038/s41584-020-0419-z PMC7178926 32327746

[71] Harmer D Falank C Reagan MR. Interleukin-6 Interweaves the Bone Marrow Microenvironment, Bone Loss, and Multiple Myeloma. Front Endocrinol (Lausanne) (2019) 9:788. doi: 10.3389/fendo.2018.00788 30671025 PMC6333051

[72] Feng W Liu B Liu D Hasegawa T Wang W Han X. Long-Term Administration of High-Fat Diet Corrects Abnormal Bone Remodeling in the Tibiae of Interleukin-6-Deficient Mice. J Histochem Cytochem (2016) 64:42–53. doi: 10.1369/0022155415611931 26416243 PMC4810790

[73] Scheidt-Nave C Bismar H Leidig-Bruckner G Woitge H Seibel MJ Ziegler R. Serum Interleukin 6 Is a Major Predictor of Bone Loss in Women Specific to the First Decade Past Menopause. J Clin Endocrinol Metab (2001) 86(5):2032–42. doi: 10.1210/jc.86.5.2032 11344203

[74] Pohin M Guesdon W Mekouo AAT Rabeony H Paris I Atanassov H. Oncostatin M Overexpression Induces Skin Inflammation But is Not Required in the Mouse Model of Imiquimod-Induced Psoriasis-Like Inflammation. Eur J Immunol (2016) 46:1737–51. doi: 10.1002/eji.201546216 27122058

[75] Sims NA Quinn JMW. Osteoimmunology: Oncostatin M as a Pleiotropic Regulator of Bone Formation and Resorption in Health and Disease. Bonekey Rep (2014) 3:527. doi: 10.1038/bonekey.2014.22 24876928 PMC4037876

[76] de Souza PPC Henning P Lerner UH. Stimulation of Osteoclast Formation by Oncostatin M and the Role of WNT16 as a Negative Feedback Regulator. Int J Mol Sci (2022) 23:3287. doi: 10.3390/ijms23063287 35328707 PMC8953253

[77] Michalak-Stoma A Bartosińska J Kowal M Raczkiewicz D Krasowska D Chodorowska G. IL-17A in the Psoriatic Patients’ Serum and Plaque Scales as Potential Marker of the Diseases Severity and Obesity. Mediators Inflammation (2020) 2020:1–9. doi: 10.1155/2020/7420823 PMC7293749 32587472

[78] Prinz JC. Human Leukocyte Antigen-Class I Alleles and the Autoreactive T Cell Response in Psoriasis Pathogenesis. Front Immunol (2018) 9:954. doi: 10.3389/fimmu.2018.00954 29760713 PMC5936982

[79] Schon MP Erpenbeck L. The Interleukin-23/Interleukin-17 Axis Links Adaptive and Innate Immunity in Psoriasis. Front Immunol (2018) 9:1323. doi: 10.3389/fimmu.2018.01323 29963046 PMC6013559

[80] Zheng Y Danilenko DM Valdez P Kasman I Eastham-Anderson J Wu J. Interleukin-22, A T(H)17 Cytokine, Mediates IL-23-Induced Dermal Inflammation and Acanthosis. Nature (2007) 445:648–51. doi: 10.1038/nature05505 17187052

[81] Rizzo HL Kagami S Phillips KG Kurtz SE Jacques SL Blauvelt A. IL-23-Mediated Psoriasis-Like Epidermal Hyperplasia is Dependent on IL-17a. JImmunol (2011) 186:1495–502. doi: 10.4049/jimmunol.1001001 21172868

[82] von Stebut E Boehncke W-H Ghoreschi K Gori T Kaya Z Thaci D. IL-17A in Psoriasis and Beyond: Cardiovascular and Metabolic Implications. Front Immunol (2020) 10:3096. doi: 10.3389/fimmu.2019.03096 32010143 PMC6974482

[83] Uluckan O Wagner EF. Role of IL-17A Signalling in Psoriasis and Associated Bone Loss. ClinExpRheumatol (2016) 34:17–20. 27586798

[84] Mentzel J Kynast T Kohlmann J Kirsten H Blüher M Simon JC. Reduced Serum Levels of Bone Formation Marker P1NP in Psoriasis. Front Med (2021) 8:730164. doi: 10.3389/fmed.2021.730164 PMC8517119 34660638

[85] Uluckan O Jimenez M Karbach S Jeschke A Grana O Keller J. Chronic Skin Inflammation Leads to Bone Loss by IL-17-Mediated Inhibition of Wnt Signaling in Osteoblasts. SciTranslMed (2016) 8:330ra37. doi: 10.1126/scitranslmed.aad8996 27089206

[86] Zhang J Fu Q Ren Z Wang Y Wang C Shen T. Changes of Serum Cytokines-Related Th1/Th2/Th17 Concentration in Patients With Postmenopausal Osteoporosis. Gynecol Endocrinol (2015) 31(3):183–90. doi: 10.3109/09513590.2014.975683 25384921

[87] Deselm CJ Takahata Y Warren J Chappel JC Khan T Li X. IL-17 Mediates Estrogen-Deficient Osteoporosis in an Act1-Dependent Manner. J Cell Biochem (2012) 113(9):2895–902. doi: 10.1002/jcb.24165 PMC3640304 22511335

[88] Le Goff B Bouvard B Lequerre T Lespessailles E Marotte H Pers Y-M. Implication of IL-17 in Bone Loss and Structural Damage in Inflammatory Rheumatic Diseases. Mediators Inflammation (2019) 2019:1–9. doi: 10.1155/2019/8659302 PMC6710740 31485194

[89] Adamopoulos IE Suzuki E Chao C-C Gorman D Adda S Maverakis E. IL-17A Gene Transfer Induces Bone Loss and Epidermal Hyperplasia Associated With Psoriatic Arthritis. Ann Rheum Dis (2015) 74:1284–92. doi: 10.1136/annrheumdis-2013-204782 PMC4229480 24567524

[90] Yago T Nanke Y Ichikawa N Kobashigawa T Mogi M Kamatani N. IL-17 Induces Osteoclastogenesis From Human Monocytes Alone in the Absence of Osteoblasts, Which is Potently Inhibited by Anti-TNF-α Antibody: A Novel Mechanism of Osteoclastogenesis by IL-17. J Cell Biochem (2009) 108(4):947–55. doi: 10.1002/jcb.22326 19728295

[91] Balani D Aeberli D Hofstetter W Seitz M. Interleukin-17A Stimulates Granulocyte-Macrophage Colony-Stimulating Factor Release by Murine Osteoblasts in the Presence of 1,25-Dihydroxyvitamin D 3 and Inhibits Murine Osteoclast Development. Vitro Arthritis Rheum (2013) 65(2):436–46. doi: 10.1002/art.37762 23124514

[92] Sprangers S Schoenmaker T Cao Y Everts V de Vries TJ. Different Blood-Borne Human Osteoclast Precursors Respond in Distinct Ways to IL-17a. J Cell Physiol (2016) 231:1249–60. doi: 10.1002/jcp.25220 26491867

[93] Mostafa WZ Hegazy RA. Vitamin D and the Skin: Focus on a Complex Relationship: A Review. J Adv Res (2013) 6(6):793–804. doi: 10.1016/j.jare.2014.01.011 PMC4642156 26644915

[94] De Martinis M Ginaldi L Sirufo MM Bassino EM De Pietro F Pioggia G. IL-33/Vitamin D Crosstalk in Psoriasis-Associated Osteoporosis. Front Immunol (2021) 11:604055. doi: 10.3389/fimmu.2020.604055 33488605 PMC7819870

[95] Lee YH Song GG. Association Between Circulating 25-Hydroxyvitamin D Levels and Psoriasis, and Correlation With Disease Severity: A Meta-Analysis. Clin Exp Dermatol (2018) 43(5):529–35. doi: 10.1111/ced.13381 29341195

[96] Cubillos S Norgauer J. Low Vitamin D-Modulated Calcium-Regulating Proteins in Psoriasis Vulgaris Plaques: S100A7 Overexpression Depends on Joint Involvement. Int J Mol Med (2016) 38:1083–92. doi: 10.3892/ijmm.2016.2718 PMC5029959 27573000

[97] Compston J. Glucocorticoid-Induced Osteoporosis: An Update. Endocrine (2018) 61:7–16. doi: 10.1007/s12020-018-1588-2 29691807 PMC5997116

[98] Egeberg A Schwarz P Harsløf T Andersen YMF Pottegård A Hallas J. Association of Potent and Very Potent Topical Corticosteroids and the Risk of Osteoporosis and Major Osteoporotic Fractures. JAMA Dermatol (2021) 157:275. doi: 10.1001/jamadermatol.2020.4968 33471030 PMC7970335

[99] Paskins Z Whittle R Abdul Sultan A Muller S Blagojevic-Bucknall M Helliwell T. Risk of Fragility Fracture Among Patients With Late-Onset Psoriasis: A UK Population-Based Study. Osteoporos Int (2018) 29:1659–64. doi: 10.1007/s00198-018-4491-z 29574516

[100] Uehara R Suzuki Y Ichikawa Y. Methotrexate (MTX) Inhibits Osteoblastic Differentiation *In Vitro*: Possible Mechanism of MTX Osteopathy. J Rheumatol (2001) 28:251–6. 11246658

[101] Muñoz-Torres M Aguado P Daudén E Carrascosa JM Rivera R. Osteoporosis and Psoriasis. Actas Dermosifiliogr (2019) 110:642–52. doi: 10.1016/j.ad.2019.02.005 31151668

[102] Yoon V Maalouf NM Sakhaee K. The Effects of Smoking on Bone Metabolism. Osteoporos Int (2012) 23(8):2081–92. doi: 10.1007/s00198-012-1940-y 22349964

[103] Armstrong AW Harskamp CT Dhillon JS Armstrong EJ. Psoriasis and Smoking: A Systematic Review and Meta-Analysis. Br J Dermatol (2014) 170:304–14. doi: 10.1111/bjd.12670 24117435

[104] Favato G. High Incidence of Smoking Habit in Psoriatic Patients. Am J Med (2008) 121:e17. doi: 10.1016/j.amjmed.2007.12.007 18374672

[105] Sampson HW. Alcohol and Other Factors Affecting Osteoporosis Risk in Women. Alcohol Res Heal (2002) 26(4):292–8. PMC6676684 12875040

[106] Chakkalakal DA. Alcohol-Induced Bone Loss and Deficient Bone Repair. Alcohol Clin Exp Res (2005) 29:2077–90. doi: 10.1097/01.alc.0000192039.21305.55 16385177

[107] Brenaut E Horreau C Pouplard C Barnetche T Paul C Richard M-A. Alcohol Consumption and Psoriasis: A Systematic Literature Review. J Eur Acad Dermatol Venereol (2013) 27:30–5. doi: 10.1111/jdv.12164 23845150

[108] Auker L Cordingley L Pye SR Griffiths CEM Young HS. What are the Barriers to Physical Activity in Patients With Chronic Plaque Psoriasis?*. Br J Dermatol (2020) 183:1094–102. doi: 10.1111/bjd.18979 PMC7754450 32107775

[109] Nowowiejska J Baran A Grabowska P Lewoc M Kaminski TW Flisiak I. Assessment of Life Quality, Stress and Physical Activity Among Patients With Psoriasis. Dermatol Ther (Heidelb) (2021) 12(2):395–406. doi: 10.1007/s13555-021-00662-1 34918196 PMC8850505

[110] Hernández JL López-Mejías R Blanco R Pina T Ruiz S Sierra I. Association of Trabecular Bone Score With Inflammation and Adiposity in Patients With Psoriasis: Effect of Adalimumab Therapy. J Osteoporos (2016) 2016:1–6. doi: 10.1155/2016/5747852 PMC4880682 27293954

[111] Osmancevic A Landin-Wilhelmsen K Larkö O Mellström D Wennberg AM Hulthén L. Risk Factors for Osteoporosis and Bone Status in Postmenopausal Women With Psoriasis Treated With UVB Therapy. Acta Derm Venereol (2008) 88(3):240–6. doi: 10.2340/00015555-0403 18480922

[112] Kocijan R Englbrecht M Haschka J Simon D Kleyer A Finzel S. Quantitative and Qualitative Changes of Bone in Psoriasis and Psoriatic Arthritis Patients. J Bone Miner Res (2015) 30:1775–83. doi: 10.1002/jbmr.2521 25827104

[113] Millard TP Antoniades L Evans AV Smith HR Spector TD Barker JNWN. Bone Mineral Density of Patients With Chronic Plaque Psoriasis. Clin Exp Dermatol (2001) 26:446–8. doi: 10.1046/j.1365-2230.2001.00855.x 11488836

[114] Modalsli EH Åsvold BO Romundstad PR Langhammer A Hoff M Forsmo S. Psoriasis, Fracture Risk and Bone Mineral Density: The HUNT Study, Norway. Br J Dermatol (2017) 176:1162–9. doi: 10.1111/bjd.15123 27718508

[115] Xia J Xie S-Y Liu K-Q Xu L Zhao P-P Gai S-R. Systemic Evaluation of the Relationship Between Psoriasis, Psoriatic Arthritis and Osteoporosis: Observational and Mendelian Randomisation Study. Ann Rheum Dis (2020) 79:1460–7. doi: 10.1136/annrheumdis-2020-217892 PMC7970448 32737104

[116] Keller JJ Kang J-H Lin H-C. Association Between Osteoporosis and Psoriasis: Results From the Longitudinal Health Insurance Database in Taiwan. Osteoporos Int (2013) 24:1835–41. doi: 10.1007/s00198-012-2185-5 23052942

[117] Kathuria P Gordon KB Silverberg JI. Association of Psoriasis and Psoriatic Arthritis With Osteoporosis and Pathological Fractures. J Am Acad Dermatol (2017) 76:1045–1053.e3. doi: 10.1016/j.jaad.2016.11.046 28314685

[118] Kuo TR Chen CH. Bone Biomarker for the Clinical Assessment of Osteoporosis: Recent Developments and Future Perspectives. BiomarkRes (2017) 5:18. doi: 10.1186/s40364-017-0097-4 PMC5436437 28529755

[119] Harter L Shin D Baker JF Takeshita J Love T Gelfand J. The Risk of Fracture Among Patients With Psoriasis, Psoriatic Arthritis and Rheumatoid Arthritis. Arthritis Rheumatol (2016) 76(5):882–5. doi: 10.1136/annrheumdis-2016-210441 PMC5384863 28093419

[120] Martinez-Lopez A Blasco-Morente G Giron-Prieto MS Arrabal-Polo MA Luque-Valenzuela M Luna-Del Castillo JDD. Linking of Psoriasis With Osteopenia and Osteoporosis: A Cross-Sectional Study. Indian J Dermatol Venereol Leprol (2019) 85(2):153–9. doi: 10.4103/ijdvl.IJDVL_831_17 30226478

[121] Ogdie A Harter L Shin D Baker J Takeshita J Choi HK. The Risk of Fracture Among Patients With Psoriatic Arthritis and Psoriasis: A Population-Based Study. Ann Rheum Dis (2017) 76:882–5. doi: 10.1136/annrheumdis-2016-210441 PMC5384863 28093419

[122] Lee JW Min C Bang CH Kwon BC Choi HG. Psoriasis is Associated With an Increased Risk of Osteoporosis: Follow-Up and Nested Case–Control Studies Using a National Sample Cohort. Osteoporos Int (2021) 32(3):529–38. doi: 10.1007/s00198-020-05724-2 33151377

[123] D’Epiro S Marocco C Salvi M Mattozzi C Luci C Macaluso L. Psoriasis and Bone Mineral Density: Implications for Long-Term Patients. J Dermatol (2014) 41:783–7. doi: 10.1111/1346-8138.12546 24990650

[124] Chen TL Lu JW Huang YW Wang JH Su KY. Bone Mineral Density, Osteoporosis, and Fracture Risk in Adult Patients With Psoriasis or Psoriatic Arthritis: A Systematic Review and Meta-Analysis of Observational Studies. J Clin Med (2020) 9(11):3712. doi: 10.3390/jcm9113712 PMC7699147 33227975

[125] Sepehri NZ Raeisi T Razi B Janmohammadi P Darand M Alizadeh S. The Association Between Psoriasis and Psoriatic Arthritis With the Risk of Osteoporosis, Osteopenia and Bone Fractures: A Systematic Review and Meta-Analysis. Int J Clin Pract (2021) 75:e14630. doi: 10.1111/ijcp.14630 34260133

[126] Pinter A Schwarz P Gerdes S Simon JC Saalbach A Rush J. Biologic Treatment in Combination With Lifestyle Intervention in Moderate to Severe Plaque Psoriasis and Concomitant Metabolic Syndrome: Rationale and Methodology of the METABOLyx Randomized Controlled Clinical Trial. Nutrients (2021) 13:3015. doi: 10.3390/nu13093015 34578893 PMC8471656

